# Supplementary material for: A Viterbi decoder and its hardware Trojan models: an FPGA-based implementation study
Source: PeerJ Comput Sci. 2020 Mar 2;6:e250. doi: 10.7717/peerj-cs.250 (PMC7924523; doi:10.7717/peerj-cs.250)
Supplement: Supplemental Information 1 — An example for viterbi decoding is illustrated with an trellis diagram [file peerj-cs-06-250-s001.docx]

**Viterbi decoding elucidated with an example**

The Viterbi decoding procedure can be demonstrated using the trellis shown below.


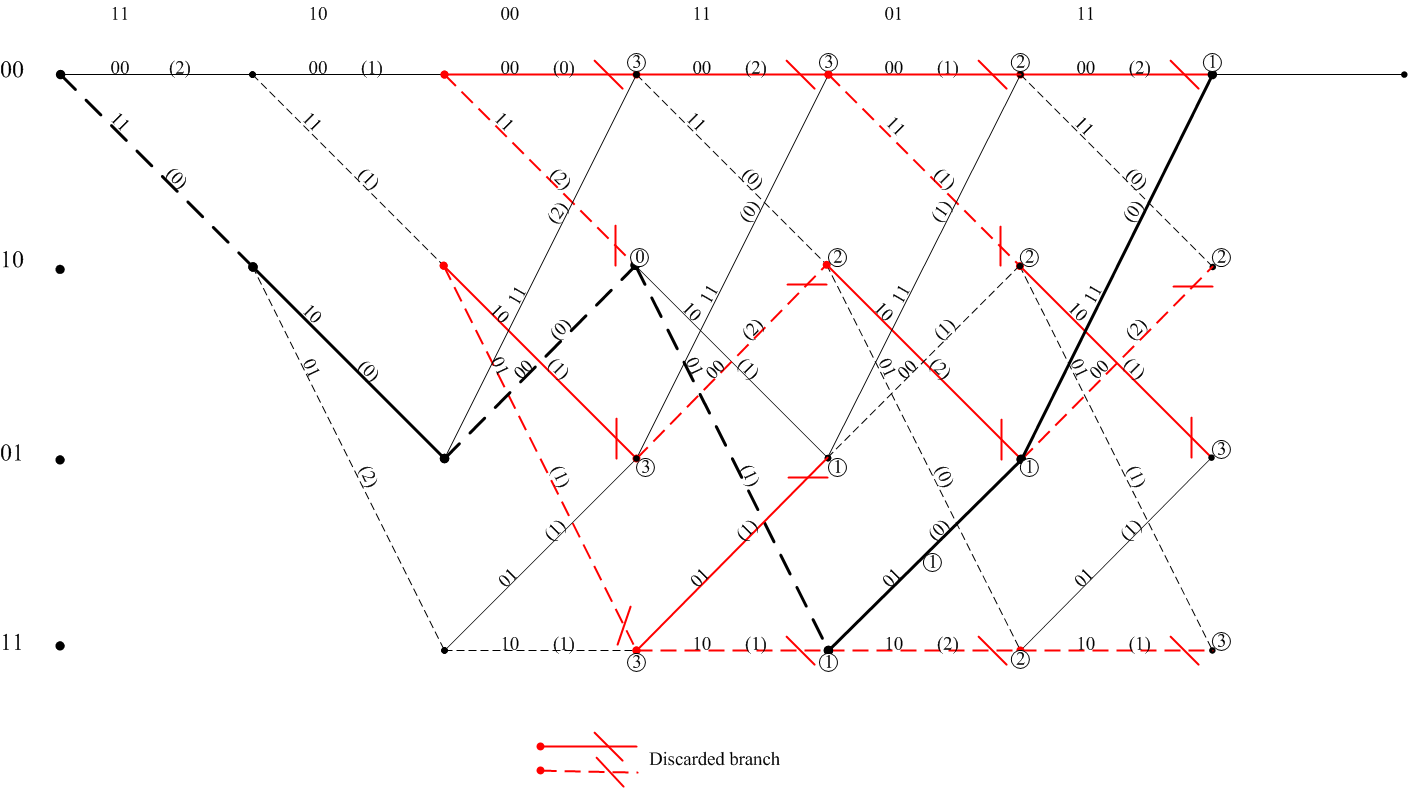


**Trellis diagram showing the decoding of the sequence 11 10 00 11 01 11 using Viterbi algorithm**

Consider a received sequence 11 10 00 11 01 11 that is input to the Viterbi decoder. The branch metric for every branch in the trellis is computed as the difference in the Hamming distance between the branches of the received sequence and the branch outputs in the trellis at each stage for all the states and are shown in parenthesis in the figure.

The sequences of branches leading to a node is called a path. When a node has more than one incoming path, selection of the path with the lowest cumulative Hamming distance path metric is to be done. The sum of the branch metrics along any path leading to a node is called the path metric. At any node, the path with the lowest path metric is retained as the survivor path, and the rest of the paths are discarded. The path metric computed for the survivor paths are shown as encircled numbers in the figure. For example at stage 3, the first node has two incoming paths each with path metrics 3 and 2 respectively. The path with metric value 2 is selected as the survivor path. This procedure of branch metric and path metric computations and survivor path selection are continued till the all zero state is reached which indicate the termination of the input sequence. The survivor path is traced back starting from the terminating all zero state to the initial all zero state. The branch words along this sequence of survivor paths traced back from the terminating state represent the decoded codeword sequence. The trace back path is shown in bold in the figure. The branch word along this trace back path is 11 10 00 01 01 11. Hence from the received sequence 11 10 00 11 01 11 the single error present is corrected and the decoded codeword is obtained as 11 10 00 01 01 11.
